# Faecal haemoglobin-based referral and investigation prioritisation is associated with colorectal cancer-specific survival in symptomatic patients: a retrospective observational study

**DOI:** 10.1038/s41416-026-03378-1

**Published:** 2026-04-02

**Authors:** Stephen T. McSorley, Paul Burton, Donna Chantler, Mark Johnstone, Brian D. Nicholson, Graham MacKay, David Mansouri, Mark Vella, Susanti Susanti, Douglas Rigg, Jack Winter, Paul Witherspoon

**Affiliations:** 1 University of Glasgow, School of Cancer Sciences, Wolfson Wohl Cancer Research Centre, Glasgow, UK; 2 Department of Coloproctology, Glasgow Royal Infirmary, NHS Greater Glasgow and Clyde, Glasgow, UK; 3 eHealth, Corporate Services, Business Intelligence, NHS Greater Glasgow and Clyde, Glasgow, UK; 4 Department of Clinical Biochemistry, NHS Greater Glasgow and Clyde, Glasgow, UK; 5 Nuffield Department of Primary Care Health Sciences, University of Oxford, Oxford, UK; 6 Department of General Surgery, Royal Alexandra Hospital, Paisley, UK; 7 Cancer Research UK Scotland Institute, Glasgow, UK; 8 NHS Greater Glasgow and Clyde, Glasgow, PCCN Clinical Lead & Lead Cancer GP, Glasgow, UK; 9 Department of Gastroenterology, Glasgow Royal Infirmary, NHS Greater Glasgow and Clyde, Glasgow, UK; 10 Department of Colorectal Surgery, Queen Elizabeth University Hospital, NHS Greater Glasgow and Clyde, Glasgow, UK

**Keywords:** Laboratory techniques and procedures, Outcomes research, Colorectal cancer

## Abstract

**Background:**

Current BSG/ACPGBI and NICE guidance recommends that faecal haemoglobin (f-Hb) ≥10 ug/g measured by faecal immunochemical test (FIT) in symptomatic patients should prompt referral through cancer prioritised diagnostic pathways. However, limited long term CRC outcome data exist. This study compared CRC specific survival (CSS) between patients by f-Hb concentration and referral priority in a large primary care f-Hb prioritised lower GI symptomatic pathway.

**Methods:**

Retrospective single health board study of symptomatic patients submitting FIT in primary care, 2019–2022. CRC diagnoses up to 3 years after pathway entry, and CRC deaths (ICD10 18, 19, 20) to end 2024 were recorded from cancer audit and MCN datasets. Patients were grouped by f-Hb concentration and referral priority. Univariable and multivariable Cox regression estimated CSS.

**Result:**

Of 126,984 patients, 1453 (1%) were diagnosed with CRC within 3 years of f-Hb result or referral, of which 444 (31%) died due to CRC. At multivariable analysis, referral without FIT (HR 1.42, 95% CI 1.06–1.91), and f-Hb ≥10 ug/g diagnosed outwith CRC prioritised pathways (HR 1.47, 95% CI 1.03–2.10) were associated with worse CSS independent of TNM stage.

**Conclusion:**

Referral and investigation through cancer prioritised pathways guided by f-Hb concentration is safe in relation to CSS.

## Introduction

Although there are established biannual faecal immunochemical test (FIT) based bowel screening programmes throughout the UK, presentation via primary care with symptoms is the commonest mode of diagnosis of colorectal cancer [1]. As such there are symptom-based colorectal cancer referral guidelines for primary care published by the National Institute for Health and Clinical Excellence (NICE) for England, Wales and Northern Ireland [2], and by Healthcare Improvement Scotland (HIS) in Scotland [3]. Patients meeting these criteria have been recommended to be referred at either ‘Urgent Suspicion of Cancer’ (USC) (Scotland) or ‘suspected cancer pathway’ (rest of UK) priority. However, it is clear that specific symptoms, including those within current guidelines, have a low positive predictive value (PPV) for colorectal cancer [4].

The use of faecal haemoglobin (f-Hb) concentration measured by FIT as a method of stratifying the likelihood of CRC in symptomatic patients presenting to primary care began in 2015 in NHS Tayside [5]. This promising initial data led to a recommendation from the NICE (DG30) that f-Hb could be considered as an adjunct to symptoms in symptomatic pathways [6]. Since then, a number of groups in the UK have published large observational datasets summarised in a meta-analysis, which was used to inform British Society of Gastroenterology (BSG) and Association of Coloproctologists of Great Britain and Ireland (ACPGBI) guidance to UK primary and secondary care clinicians involved in the care of patients presenting with symptoms concerning for colorectal cancer [7]. These guidelines recommend that f-Hb concentrations ≥10 ug/g in patients with new persistent bowel symptoms should be used to guide referral for assessment and investigation at USC or suspected cancer pathway priority, while concentrations <10 ug/g could allow for referral at a lower priority or ongoing symptomatic management in primary care. This guidance was followed by firmer guidance from NICE recommending the use of f-Hb with the same threshold of 10 ug/g to guide referral along a suspected cancer pathway in those with new persistent symptoms [8].

Recently a number of studies have examined the combination of FIT and routine demographic data [9], components of full blood count such as Hb and platelet count [10], combined laboratory assessment of iron deficiency anaemia [11, 12], and the use of a repeat FIT [13, 14], all of which have shown promise in terms of predictive values for CRC. There have also been calls to increase the f-Hb threshold for referral to a cancer prioritised pathway to 20 ug/g as the PPV for CRC does not appear to exceed 3% below this threshold, and to improve the current demand/capacity mismatch in endoscopy and diagnostics services [12, 15].

However, data on long-term outcomes in those diagnosed with CRC through FIT-based pathways remain lacking. It is recognised that the use of any f-Hb threshold will result in ‘false negatives’, patients diagnosed with CRC despite having f-Hb levels lower than the value specified in the pathway. The reported rate of this at a f-Hb threshold of 10 ug/g is around 10% [16], and factors associated with so-called ‘FIT negative’ CRC have been described, including proximal lesions, obstructing lesions and sex [17]. The concern in this group of patients is that having f-Hb <10 ug/g, or in future <20 ug/g, may lead to diagnostic delay, higher stage at the time of eventual diagnosis, and so poorer long-term outcomes. The counter argument is that the improved resource use in the majority of CRC patients who have f-Hb above the threshold will improve outcomes at the population level.

The aim of this study, therefore, was to examine patients submitting FIT via primary care and referred through a large f-Hb prioritised lower GI symptomatic pathway, comparing CRC-specific survival by f-Hb group and referral priority.

## Methods

### Pathway

NHS Greater Glasgow and Clyde (NHSGGC) introduced primary care initiated FIT into symptomatic lower GI referral pathways in August 2018 [18]. Primary care clinicians were asked to request a FIT to be submitted by all patients with any new persistent lower GI symptoms. Key exceptions to this were a palpable abdominal, anal or rectal mass found on clinical examination, or asymptomatic iron deficiency anaemia directing referral at USC priority without the need for f-Hb (Supplementary Table 1). The pathway otherwise adopted NICE recommendations to prioritise referrals to secondary care with f-Hb ≥10 ug/g as USC, and those <10 ug/g as Urgent, Routine, or managed in primary care (Supplementary Fig. 1). In addition, f-Hb concentrations were used to further prioritise secondary care investigation with those >399 ug/g deemed highest priority (‘Category 1’), followed by those 80–399 ug/g (‘Category 2’) and then those 10–79 ug/g (‘Category 3’). Those patients referred with f-Hb <10 ug/g felt to require further investigation were investigated at Category 4 priority.

FIT specimen collection devices (EXTEL HEMO-AUTO MC, Minaris Medical America, USA) were supplied to all NHSGGC GP practices with written and pictorial instructions for practitioners and patients in multiple languages, and return envelopes. Kits were transported at ambient temperatures through routine specimen collection services and stored at 4 °C before being analysed using the HM-JACKarc platform (Hitachi Chemical Diagnostics Systems, Japan). The manufacturers quote a f-Hb limit of detection of 2 µg/g, a limit of quantification of 7 µg/g and an upper measurement limit of 400 µg/g. Specimens with f-Hb concentrations above this limit were not diluted and re-analysed. Analysis was carried out Monday to Friday. The f-Hb result was reported electronically to the requesting GP, with a normal reference range of ≤9 ug/g and with advice to refer at USC priority to colorectal surgery when ≥10 ug/g.

At the point of referral vetting in secondary care patients with f-Hb ≥10 ug/g were where possible sent direct to test with colonoscopy. CT pneumocolon, CT abdomen pelvis, CT thorax abdomen pelvis or attendance at colorectal surgery or gastroenterology clinic were requested for those who the vetting clinician regarded this to be more appropriate based on history, co-morbidity or medication in the electronic referral and/or electronic health record.

### Patient cohort

The study included patients aged 16 years or older within NHSGGC who between December 2018 and January 2023 submitted a FIT requested in primary care and were not subsequently referred to colorectal surgery or gastroenterology services at any priority; submitted a FIT requested in primary care and were then referred to colorectal surgery or gastroenterology services via the symptomatic pathway at any priority; and patients who were referred to colorectal surgery or via the symptomatic pathway at USC priority without a FIT request.

If more than one FIT sample had been submitted, then the f-Hb with the highest concentration was used in analysis. Similarly, if more than one referral had been made to colorectal surgery or gastroenterology, the referral with the highest priority was used in analysis. Patients diagnosed with CRC and not referred via the colorectal surgery or gastroenterology pathway were linked to referrals through other specialties and ED attendances 60 days either side of that date to capture emergency presentation of patients who had submitted FIT within the last 3 years of diagnosis. Patients entering the symptomatic pathway or having a primary care FIT requested, who had undergone previous treatment for CRC were included; however, only a de novo/metachronous CRC diagnosis within 3 years of FIT or referral was considered within the outcome recording.

### Data linkage, collection and definitions

The unique Community Health Index (CHI) number identifier was used as the linkage variable throughout.

All FIT requests received by NHSGGC were retrieved from the laboratory information management system (iLab TP v1.9, Telepath) and all referrals to colorectal surgery or gastroenterology via the Scottish national online referral management system SCI Gateway (Scottish Care Information Gateway R.20) between December 2018 and January 2023.

Patient age at FIT submission or referral, sex, the presence of iron deficiency anaemia (IDA), and postcode were collected through the secure NHS GGC patient information repository SCI Store (Scottish Care Information Store). Anaemia was defined per WHO guidelines as haemoglobin (Hb) concentrations <130 mg/L in males or <120 mg/L in females and iron-deficient (IDA) if ferritin <30 ug/L [19]. Postcode was used to determine the patient’s Scottish Index of Multiple Deprivation (SIMD), a measure of a geographical area’s deprivation according to education, employment, income, health, housing, crime and service access (SIMD 2020).

Unisoft (Unisoft GI Reporting Software, v2.5, Unisoft Medical Systems, UK) was used to identify patients who underwent colonoscopy, and CRIS (Central Data Networks Radiology Information System) patients who underwent CT pneumocolon, CT abdomen pelvis or CT thorax abdomen pelvis. ‘Time to ‘scope’ and ‘time to CT scan” were defined as days between date of submission of f-Hb with the highest result, or referral to colorectal surgery or gastroenterology with highest priority, and the date of the test, but not necessarily the result. Up to 30 days before the date of FIT or referral were included in the time range to allow for delays in FIT processing and result checking in cases where, for example, a referral had been made simultaneously with the FIT being provided to a patient.

Local cancer audit data and the Managed Clinical Network (MCN) cancer registry were used to identify patients diagnosed with CRC within 3 years of FIT submission with highest f-Hb or referral at the highest priority using ICD10 codes 18, 19 and 20, including the date of diagnosis, TNM stage and primary tumour location. The total diagnostic interval was defined as the time between the date of maximum f-Hb result or referral and the recorded date of CRC diagnosis [20]. The date of CRC diagnosis was recorded based on either histological or radiological results, whichever came first. Again, up to 30 days before the date of FIT or referral were included in the time range. Attendances at the Emergency Department (ED) were recorded 60 days before or after the date of CRC diagnosis.

Local cancer audit data, MCN data and Scottish Morbidity Record (SMR) were used to identify those patients who had died, their date of death and cause of death as CRC-specific (ICD10 codes 18, 19 and 20) or other (all other ICD10 codes). Cancer-specific survival (CSS) was defined as the time from the date of CRC diagnosis to CRC-specific death in days. The date of censor was 31st January 2025.

### Statistical analysis

Patients were grouped by f-Hb category; <10 ug/g, 10–79 ug/g, 80–399 ug/g, >399 ug/g, f-Hb submitted but with no valid result available (due to spoiled kit/user error), and f-Hb not submitted. Variables were compared across the groups at the univariable level using the chi-squared test for categorical variables presented as number and percentage proportion, and Kruskal–Wallis test for continuous variables presented as median and interquartile range (IQR). *P* values were considered statistically significant if <0.05 after adjustment for multiple testing using Benjamini-Hochberg correction.

For patients diagnosed with CRC, Cox regression was used for survival analysis with reference to CSS, with variables with at least one statistically significant category at univariable analysis entered into a multivariable model where *p* values < 0.05 were considered statistically significant. No conditional removal was used as it was felt that the model should always include specific variables—namely f-Hb concentration group, referral priority and TNM stage.

To further assess the impact of f-Hb associated referral priority on CSS, patients diagnosed with CRC were then re-grouped around the guideline f-Hb threshold of 10 ug/g and referral category into; f-Hb ≥10 ug/g and USC, f-Hb ≥10 ug/g and Urgent/Routine, f-Hb ≥ 10 ug/g and ED/other specialty, f-Hb < 10 ug/g and USC, f-Hb <10 ug/g and Urgent/Routine, f-Hb <10 ug/g and ED/other specialty. Similar comparison of patient and pathway variables, univariable and multivariable survival analyses were undertaken.

All statistical analyses were performed in SPSS (v29.0.1.0, IBM, NY, USA).

### Ethics approval and consent

The data linkage, collection, retention, processing and analysis was approved by the NHS Greater Glasgow and Clyde Caldicott Guardian and Information Governance Officer as part of the CRUK TET project, which was considered service evaluation and development, without the need for a full ethics committee application or individual patient consent.

## Results

### Patients

**Fig. 1:**
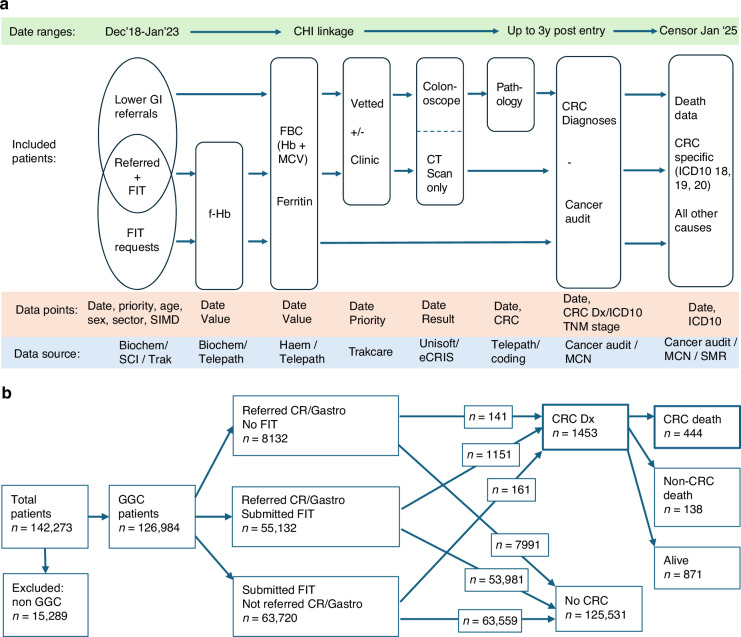
Schematic representation of study data collection and patient flow. **a** Patient identification and data collection of included patients. **b** Flow of identified patients through the NHS Greater Glasgow and Clyde lower GI symptomatic pathway to CRC diagnosis and death. CHI Community Health Index, CR colorectal surgery, CRC colorectal cancer, CRIS Central Data Networks Radiology Information System, FIT faecal immunochemical test, f-Hb faecal haemoglobin, MCN managed clinical network, MCV mean corpuscular volume, SCI Scottish Care Information, SIMD Scottish index of multiple deprivation, SMR Scottish morbidity record.

After the exclusion of 15,289 patients who lived outside of NHSGGC but had f-Hb samples processed in Glasgow, 126,984 eligible patients during the time period December 2018–January 2023 were investigated (Fig. 1). 54,113 (43%) were male and 72,871 (57%) were female. Their median age was 59 years (IQR 45–71). 8132 (7%) were referred to colorectal surgery or via the symptomatic pathway at USC priority *without* a FIT request. 118,852 (93%) patients submitted at least one FIT, of which 55,132 (43%) were then referred to colorectal surgery or gastroenterology services via the symptomatic pathway, while 63,720 (50%) were *not* subsequently referred to colorectal surgery or gastroenterology services at any priority. 1453 (1%) were diagnosed with CRC within 3 years of the date of f-Hb result or referral (highest value and highest priority if more than one). Of those patients, 444 (31%) died due to CRC, and 138 (9%) due to other causes within the study period, with a median follow-up time of those alive at censoring of 39 months (IQR 30–52).

### Temporal trends in referral and FIT submission

Comparing the four complete years 2019–2022, the number of patients with a primary care requested FIT rose from 16,316 in 2019, to 47,995 in 2022, with a fall in those having no valid associated f-Hb result from 3828 (20%) to 2862 (6%)(*p*_adj_ <0.001). The number of patients submitting 2 or more FITs within a 12-month period increased from 1153 (7%) in 2019 to 5020 (10%) (*p*_adj_ <0.001). There was no clinically significant difference in the positivity rate of FIT (maximum value for those submitting >1 within a 12 month period) at a f-Hb threshold of 10 ug/g between 2019 (23%) and 2022 (24%). The number of referrals to colorectal surgery or gastroenterology increased from 10,465 in 2019 to 23,899 in 2022, with an increase in USC referrals from 5336 (51%) to 14,118 (59%)(*p* < 0.001). The CRC diagnostic rate was 1.4% (*n* = 268) in patients entering the pathway in 2019, and although the number of CRCs diagnosed in those entering in 2022 increased to 449, the increase in submitted FIT and referrals meant that the CRC diagnostic rate fell to 0.9% (p < 0.001).

### Primary care-based FIT prioritises referral, investigation and stratifies CRC diagnostic rate in symptomatic patients

Of all included patients (Table 1), those who had submitted a FIT but with no valid f-Hb results (*n* = 3573) were most likely to have no recorded referral to colorectal surgery or gastroenterology (87%), followed by those with f-Hb <10 ug/g (65%), while only 9% of those with f-Hb > 399 ug/g were not referred (*p*_adj_ < 0.001). Conversely, those with f-Hb >399 ug/g were most likely to be referred at USC priority (68%), while those with f-Hb <10 ug/g (12%) and submitted but unknown f-Hb (5%) were least likely. Likewise, those with f-Hb >399 ug/g were most likely to attend OPD (p_adj_ <0.001), have a lower GI endoscopy (p_adj_ <0.001), or CT (p_adj_ <0.001) within 3 years of maximum f-Hb result or referral, with those who had submitted a FIT but with no valid f-Hb result being least likely.

**Table 1:** Patient characteristics, pathway process measurement and colorectal cancer detection rate by highest faecal haemoglobin concentration result in all patients submitting faecal immunochemical test in primary care, or referred to lower GI secondary care services in NHS Greater and Glasgow and Clyde, December 2018 to January 2023.

		Faecal immunochemical test faecal haemoglobin (f-Hb) concentration (ug/g)							
		>399	80–399	10–79	<10	Submitted no valid result	Not submitted	*p*	*p*_adj_
Patients	*n* (%)	6489 (5)	5269 (4)	15516 (12)	88,005 (69)	3573 (3)	8132 (7)	−	−
Patient characteristics	(% within rows)								
Age (yrs)	Median (IQR)	61 (44–74)	65 (51–76)	66 (54–76)	57 (43–69)	61 (46–75)	63 (52–73)	<0.001	<0.001
Sex n(%)	M	3292 (6)	2405 (4)	6781 (13)	36,293 (67)	1725 (3)	3617 (7)	<0.001	<0.001
	F	3197 (4)	2864 (4)	8735 (12)	51,712 (71)	1848 (3)	4515 (6)		
SIMD quintile n(%)	1 (most deprived)	2377 (6)	1907 (5)	5661 (13)	28,103 (67)	597 (1)	3356 (8)	<0.001	<0.001
	2	1233 (6)	1003 (4)	2947 (13)	15,988 (69)	272 (1)	1595 (7)		
	3	835 (6)	629 (4)	1849 (12)	11,046 (71)	178 (1)	933 (6)		
	4	868 (5)	706 (4)	2127 (12)	12,773 (73)	171 (1)	945 (5)		
	5 (least deprived)	1128 (4)	979 (4)	2809 (11)	19,175 (75)	188 (1)	1277 (5)		
Year of entry to pathway n(%)	2019	707 (4)	654 (3)	2070 (11)	11,699 (62)	1186 (6)	2642 (14)	<0.001	<0.001
	2020	1220 (6)	893 (4)	2524 (12)	14,491 (67)	625 (3)	1665 (8)		
	2021	1988 (6)	1577 (4)	4414 (12)	25,936 (71)	873 (2)	1852 (5)		
	2022	2574 (5)	2145 (4)	6508 (13)	35,879 (72)	889 (2)	1973 (4)		
Anaemia n(%)^a^	No	4002 (72)	3115 (69)	9439 (70)	57,661 (81)	805 (71)	4880 (75)	<0.001	<0.001
	Yes – not IDA^b^	776 (14)	785 (17)	2073 (16)	6572 (9)	197 (17)	887 (14)		
	Yes – IDA^b^	808 (14)	650 (14)	1896 (14)	6590 (9)	140 (12)	768 (12)		
Pathway characteristics	(% within columns)								
Referral^c^ priority n(%)	USC	4405 (68)	3331 (63)	8693 (56)	10,616 (12)	192 (5)	8132 (100)	<0.001	<0.001
	Urgent	1120 (17)	1071 (20)	3735 (24)	7506 (9)	130 (4)	−		
	Routine	356 (6)	305 (6)	1184 (8)	12356 (14)	132 (4)	−		
	None/not recorded	608 (9)	562 (11)	1904 (12)	57527 (65)	3119 (87)	−		
OPD within 3 years^d^	No	1722 (27)	1731 (33)	5864 (38)	54,540 (62)	3094 (86)	2817 (35)	<0.001	<0.001
	Yes	4767 (74)	3538 (67)	9652 (62)	33465 (38)	479 (13)	5315 (65)		
Time to OPD (days)^e^	Median (IQR)	17 (8–43)	20 (9–49)	24 (10–56)	46 (14–163)	33 (9–119)	20 (10–46)	<0.001	<0.001
Scope within 3 years^d^	No	1360 (21)	1550 (29)	7450 (48)	76604 (87)	3346 (94)	6536 (80)	<0.001	<0.001
	Yes	5129 (79)	3719 (71)	8066 (52)	11401 (13)	227 (6)	1596 (20)		
Time to scope (days)^e^	Median (IQR)	28 (21–43)	38 (25–64)	58 (34–124)	218 (65–564)	66 (29–267)	31 (16–94)	<0.001	<0.001
CT scan^df^ within 3 years^d^	No	4083 (63)	3378 (64)	10,573 (68)	73,868 (84)	3251 (91)	5515 (68)	<0.001	<0.001
	Yes	2406 (37)	1891 (36)	4943 (32)	14137 (16)	322 (9)	2617 (32)		
Time to CT scan^f^ (days)^e^	Median (IQR)	71 (40–224)	87 (45–230)	114 (51–320)	203 (59–562)	104 (30–454)	43 (23–160)	<0.001	<0.001
CRC diagnosis within 3 years n(%)^g^	No	5866 (90)	5018 (95)	15,259 (98)	87842 (99.8)	3555 (99.5)	7991 (98)	<0.001	<0.001
	Yes	623 (10)	251 (5)	257 (2)	163 (0.2)	18 (0.5)	141 (2)		

*CRC* colorectal cancer, *CT* computed tomography scan, *F* female, *f-Hb* faecal haemoglobin, *FIT* faecal immunochemical test, *IQR* interquartile range, *M* male, *OPD* outpatient department, *SIMD* Scottish Index of Multiple Deprivation, *USC* urgent suspicion of cancer.

^a^M: Hb <130 mg/L, F: Hb<120 mg/L.

^b^Anaemia and ferritin <30 ug/L.

^c^Primary care referral to Colorectal Surgery or Gastroenterology.

^d^30 days before to 1095 days after submission of FIT (highest f-Hb concentration if >1 within 365 days) or referral and OPD appointment or test.

^e^Defined as time in days between date of maximum f-Hb result or referral and date of OPD or investigation appointment.

^f^CT pneumocolon, CT thorax and abdomen and pelvis, or CT abdomen and pelvis.

^g^CRC diagnosis date recorded in cancer audit data between 30 days before and 1095 days after submission of FIT or referral (ICD10 codes C18, C19, C20).

The rate of CRC diagnosed within 3 years of maximum f-Hb result stratified from 10% (*n* = 623) in those with f-Hb >399 ug/g to 0.2% (*n* = 163) in those with f-Hb <10 ug/g. Using a f-Hb threshold for investigation of 10 ug/g would have resulted in a false negative rate (FNR) of 12.6%, a positive predictive value (PPV) of 4.1% and number needed to scope (NNS) of 24 for CRC (Supplementary Table 2). Increasing this to a f-Hb threshold of 20 ug/g would have resulted in FNR of 16.8%, PPV 4.8% and NNS of 21 for CRC.

### In patients diagnosed with CRC, referral without FIT is associated with worse CSS despite USC referral priority and shorter time to diagnosis

Of the 1453 patients diagnosed with CRC (Table 2), those with f-Hb <10 ug/g had the longest times to investigation and the longest total diagnostic interval (median 176 days, IQR 56–571) with those f-Hb >399 ug/g (median 29 days, IQR 21–54) and with no submitted FIT (all of whom were referred at USC priority) being shortest (median 28 days, IQR 18–48) (*p*_adj_ <0.001).

**Table 2:** Patient characteristics, pathway process measurement, stage and survival in patients diagnosed with colorectal cancer within 3 years of submitting faecal immunochemical test in primary care, or being referred to lower GI secondary care services in NHS Greater and Glasgow and Clyde December 2018 to January 2023.

		Faecal immunochemical test faecal haemoglobin (f-Hb) concentration (ug/g)							
		>399	80–399	10–79	<10	Submitted no valid result	Not submitted	*p*	*p* adj
Patients	n(%)	623 (43)	251 (17)	257 (18)	163 (11)	18 (1)	141 (10)	−	−
Patient characteristics	(% within rows)								
Age (yrs)	Median (IQR)	69 (60–79)	70 (61–81)	73 (64–80)	70 (62–79)	74 (61–82)	72 (65–81)	0.038	0.038
Sex n(%)	M	394 (47)	132 (16)	136 (16)	80 (10)	13 (2)	74 (9)	<0.001	0.001
	F	229 (37)	119 (19)	121 (19)	83 (13)	5 (1)	67 (11)		
SIMD quintile *n*(%)	1 (most deprived)	191 (41)	72 (15)	84 (18)	56 (12)	7 (1)	59 (13)	0.011	0.012
	2	113 (40)	54 (19)	38 (14)	43 (15)	2 (1)	32 (11)		
	3	81 (45)	37 (21)	26 (15)	14 (8)	1 (1)	19 (11)		
	4	94 (46)	38 (19)	37 (18)	19 (9)	4 (2)	13 (6)		
	5 (least deprived)	143 (45)	50 (16)	72 (23)	31 (10)	4 (1)	17 (5)		
Year of entry to pathway *n*(%)	2019	85 (32)	46 (17)	43 (16)	24 (9)	7 (3)	63 (23)	<0.001	<0.001
	2020	128 (40)	61 (19)	57 (18)	40 (12)	5 (2)	28 (9)		
	2021	192 (46)	66 (16)	74 (18)	57 (13)	3 (1)	25 (6)		
	2022	218 (49)	78 (17)	83 (18)	42 (9)	3 (1)	25 (6)		
Anaemia *n* (%)^a^	No	319 (55)	138 (59)	123 (52)	84 (55)	7 (44)	55 (44)	0.214	0.338
	Yes – not IDA^b^	101 (18)	35 (15)	45 (19)	30 (20)	6 (37)	26 (21)		
	Yes – IDA^b^	153 (27)	61 (26)	69 (29)	38 (25)	3 (19)	43 (35)		
Pathway characteristics	(% within columns)								
Referral^c^/presentation *n* (%)	USC	499 (80)	184 (74)	174 (68)	59 (36)	8 (44)	141 (100)	<0.001	<0.001
	Urgent	56 (9)	46 (18)	44 (17)	22 (13)	4 (22)	0 (0)		
	Routine	25 (4)	5 (2)	10 (4)	14 (9)	1 (6)	0 (0)		
	ED^d^ – no GP ref	12 (2)	8 (3)	16 (6)	28 (17)	3 (17)	0 (0)		
	Other/Not recorded	31 (5)	8 (3)	13 (5)	40 (25)	2 (11)	0 (0)		
Time to OPD (days)^e^	Median (IQR)	25 (11–51)	30 (13–56)	41 (14–98)	47 (18–171)	39 (24–148)	20 (11–42)	<0.001	<0.001
Time to scope (days)^e^	Median (IQR)	28 (20–48)	38 (24–63)	73 (31–190)	206 (68–642)	55 (26–133)	35 (21–67)	<0.001	<0.001
Time to CT scan^f^ (days)^e^	Median (IQR)	44 (30–67)	55 (35–88)	69 (40–143)	124 (41–304)	66 (20–230)	33 (20–49)	<0.001	<0.001
Total diagnostic interval (days)^g^	Median (IQR)	29 (21–54)	41 (25–77)	68 (29–152)	176 (56–571)	61 (26–174)	28 (18–48)	<0.001	<0.001
Tumour characteristics	(% within columns)								
TNM stage n(%)	1	91 (15)	47 (19)	71 (28)	34 (21)	1 (5)	11 (8)	<0.001	<0.001
	2	149 (24)	62 (24)	56 (22)	30 (19)	3 (17)	26 (18)		
	3	166 (26)	60 (24)	55 (21)	35 (21)	2 (11)	40 (28)		
	4	136 (22)	53 (21)	47 (18)	46 (28)	9 (50)	39 (28)		
	Incomplete	81 (13)	29 (12)	28 (11)	18 (11)	3 (17)	25 (18)		
Tumour location n(%)	Right colon	183 (30)	97 (39)	118 (46)	86 (53)	8 (45)	62 (44)	<0.001	<0.001
	Left colon	211 (33)	72 (28)	62 (24)	42 (25)	1 (5)	33 (23)		
	Rectum	229 (37)	81 (32)	75 (29)	34 (21)	9 (50)	45 (32)		
	Unspecified	0 (0)	1 (1)	2 (1)	1 (1)	0 (0)	1 (1)		

*CRC* colorectal cancer, *CSS* cancer-specific survival, *CT* computed tomography scan, *ED* emergency department, *F* female, *f-Hb* faecal haemoglobin, *FIT* faecal immunochemical test, *IDA* iron deficiency anaemia, *IQR* interquartile range, *M* male, *OPD* outpatient department, *SIMD* Scottish Index of Multiple Deprivation, *SE* standard Error, *USC* urgent suspicion of cancer.

^a^M: Hb <130 mg/L F: Hb<120 mg/L.

^b^Anaemia and ferritin <30 ug/L.

^c^Referral to Colorectal Surgery or Gastroenterology.

^d^ED attendance 60 days before or 60 days after the recorded date of CRC diagnosis in audit data (ICD10 codes C18, C19, C20).

^e^Defined as time in days between date of maximum f-Hb result or referral and date of OPD or investigation appointment.

^f^CT pneumocolon, CT thorax and abdomen and pelvis, or CT abdomen and pelvis.

^g^Defined as time between date of maximum f-Hb result or referral to CRC diagnosis date recorded in cancer audit data (ICD10 codes C18, C19, C20).

**Fig. 2:**
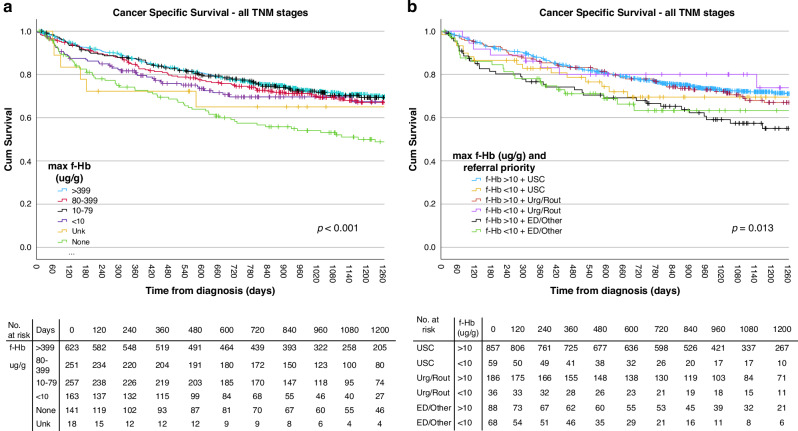
Kaplan–Meier curves of colorectal cancer (CRC) specific survival (CSS) defined by ICD10 codes 18, 19, and 20. **a** Patients with CRC grouped by faecal haemoglobin (f-Hb) concentration (ug/g), showing poorer survival (p<0.001) in those referred without a submitted faecal immunochemical test (FIT). **b** Patients with CRC grouped by f-Hb concentration (ug/g) and referral priority showing poorer survival (*p*= 0.013) in those with f-Hb ≥10 ug/g not referred to colorectal surgery or gastroenterology. USC urgent suspicion of cancer, Urg/Rout urgent or routine, ED Emergency Department, *p* by Log rank test.

The univariable CSS (Fig. 2A) was lowest in those with no submitted FIT and highest in those with f-Hb >399 ug/g (*p* < 0.001). At multivariable analysis (Table 3), referral without submitted FIT (HR 1.42, 95% CI 1.06–1.91, *p* = 0.019), and ED attendance without having been referred through primary care (HR 2.07, 95% CI 1.41–3.04, *p* < 0.001), were significantly associated with worse CSS independent of TNM stage, age, sex, and deprivation. There was no significant association between diagnostic time and CSS.

**Table 3:** Cancer Specific Survival—univariable and multivariable survival analysis in patients diagnosed with colorectal cancer within 3 years of submitting faecal immunochemical test in primary care, or being referred to lower GI secondary care services in NHS Greater and Glasgow and Clyde December 2018 to January 2023.

		Univariable HR (95% CI)	*p*	Multivariable HR (95% CI)	*p*
Age	(Years)	1.03 (1.02–1.04)	<0.001	1.03 (1.02–1.04)	<0.001
Sex	F (ref)	ref	ref	ref	ref
	M	0.92 (0.76–1.11)	0.362	−	−
SIMD quintile	1 (most deprived)	ref	ref	ref	ref
	2	1.17 (0.91–1.52)	0.223	−	−
	3	1.02 (0.75–1.39)	0.905	−	−
	4	0.98 (0.73–1.33)	0.914	−	−
	5 (least deprived)	0.86 (0.65–1.12)	0.251	−	−
IDA^a^	No (ref)	ref	ref	ref	ref
	Yes	1.10 (0.89–1.36)	0.395	−	−
f-Hb (ug/g)	>399 (ref)	ref	ref	ref	ref
	80–399	1.09 (0.83–1.42)	0.539	1.12 (0.85–1.47)	0.411
	10–79	0.98 (0.74–1.29)	0.861	1.06 (0.80–1.41)	0.670
	<10	1.22 (0.88–1.70)	0.227	1.03 (0.72–1.46)	0.889
	Submitted no valid result	1.42 (0.63–3.20)	0.400	0.72 (0.32–1.63)	0.426
	Not submitted	1.96 (1.48–2.60)	<0.001	1.42 (1.06–1.91)	0.019
Date of pathway entry	Calendar quarter^b^	0.95 (0.93–0.97)	<0.001	0.96 (0.94–0.99)	0.002
Referral^c^ / presentation	USC	ref	ref	ref	ref
	Urgent	1.05 (0.79–1.40)	0.737	1.24 (0.91–1.67)	0.169
	Routine	0.68 (0.38–1.21)	0.192	0.71 (0.40–1.28)	0.260
	ED^d^ – no GP ref	2.43 (1.70–3.48)	<0.001	2.07 (1.41–3.04)	<0.001
	Other/not recorded	0.94 (0.62–1.43)	0.783	0.96 (0.62–1.48)	0.850
Diagnostic time^e^	(Days)	1.00 (1.00–1.00)	0.716	−	−
TNM stage	1 (ref)	ref	ref	ref	ref
	2	3.23 (1.42–7.37)	0.005	2.96 (1.29–6.77)	0.010
	3	8.27 (3.81–17.93)	<0.001	9.03 (4.15–19.62)	<0.001
	4	52.81 (24.86–112.16)	<0.001	59.33 (27.83–126.48)	<0.001
	Incomplete	36.85 (17.12–79.32)	<0.001	25.35 (11.71–54.90)	<0.001

*CI* confidence interval, *ED* Emergency Department, *F* female, *FIT* faecal immunochemical test, *f-Hb* faecal haemoglobin, *HR* hazard ratio, *IDA* iron deficiency anaemia, *M* male, *SIMD* Scottish index of multiple deprivation, *USC* Urgent Suspicion of Cancer.

^a^M: Hb <130 mg/L F: Hb<120 mg/L and ferritin <30 ug/L.

^b^Calendar quarters from quarter 4 of 2018 to quarter 1 of 2023 given ascending numerical value for regression.

^c^Referral to Colorectal Surgery or Gastroenterology.

^d^ED attendance 60 days before or 60 days after recorded date of CRC diagnosis in audit data (ICD10 codes C18, C19, C20).

^e^Defined as time between date of maximum f-Hb result or referral to CRC diagnosis date recorded in cancer audit data (ICD10 codes C18, C19, C20).

Importantly, of the 141 patients diagnosed with CRC referred without FIT, only 29 (21%) had been referred without FIT with symptoms in which the pathway required it. The remainder presented with symptoms for which FIT was considered not required by the pathway (*n* = 19, 13%), or with a palpable abdominal, rectal or anal mass (*n* = 37, 26%), or in other situations for which FIT was not required (Supplementary Table 3).

In addition, there was no statistically significant difference (*p*_adj_ = 0.055) in the proportion of patients with IDA when those referred at different referral priorities with f-Hb<10 ug/g or f-Hb ≥10 ug/g were compared, in keeping with the clinical pathway of the time, which did not include IDA.

Furthermore, 77 patients diagnosed with CRC (6% of all CRC in patients with at least 1 valid f-Hb result) had more than one valid FIT test submitted prior to diagnosis, of which 8 (0.6%) had both f-Hb concentrations <10 ug/g and 22 (1.7%) had a first f-Hb <10 ug/g, with a subsequent result ≥10 ug/g. Those with both f-Hb <10 ug/g had the lowest proportion of USC referral (37% vs 68%) and the longest median diagnostic time (221 vs 178 days), small numbers prevented meaningful statistical inference or survival analysis to compare these subgroups.

### In patients diagnosed with CRC within 3 years of a valid FIT result, diagnosis outside of the appropriate f-Hb guided referral pathway and urgency are associated with worse CSS

Of the patients diagnosed with CRC, 1131 had f-Hb ≥10 ug/g and 163 <10 ug/g (Supplementary Table 4). Those with f-Hb <10 ug/g had consistently longer times to investigation and longer total diagnostic interval, with those f-Hb <10 ug/g diagnosed outwith the colorectal surgery/gastroenterology symptomatic pathway being longest (median 280 days, IQR 84–657) (*p*_adj_ < 0.001). While there was no significant difference in univariable CSS when those with f-Hb <10 ug/g were compared to those with f-Hb ≥10 ug/g (*p* = 0.227), there was a non-significant trend toward poorer CSS within the first 2 years after diagnosis in the former, with survival curves crossing at 3 years (Supplementary Fig. 2A). There was a higher proportion of patients with TNM stage 4 disease in those referral groups with f-Hb <10 ug/g (25–29%) compared to those with f-Hb ≥10 ug/g (20–25%), however this also did not reach statistical significance (*p*_adj_=0.226) Furthermore, at multivariable analysis none of the f-Hb <10 ug/g referral subgroups had significantly poorer CSS when compared to those with f-Hb ≥10 ug/g referred at USC priority (Table 4).

**Table 4:** Cancer-specific survival—univariable and multivariable survival analysis in patients diagnosed with colorectal cancer within 3 years of submitting faecal immunochemical test in primary care, or being referred to lower GI secondary care services in NHS Greater and Glasgow and Clyde December 2018 to January 2023.

		Univariable HR (95% CI)	*p*	Multivariable HR (95% CI)	*p*
Age	(Years)	1.03 (1.02–1.04)	<0.001	1.03 (1.02–1.04)	<0.001
Sex	F (ref)	ref	ref	ref	ref
	M	0.92 (0.75–1.13)	0.442	−	−
Max f-Hb (ug/g) and	≥10 + USC	ref	ref	ref	ref
Referral^a^ priority	≥10 + Urgent/Routine	1.12 (0.84–1.49)	0.454	1.19 (0.88–1.60)	0.261
	≥10 + ED/other	1.70 (1.19–2.42)	0.003	1.47 (1.03–2.10)	0.036
	<10 + USC	1.25 (0.75–2.07)	0.396	1.20 (0.72–2.00)	0.491
	<10 + Urgent/Routine	0.86 (0.43–1.75)	0.681	0.77 (0.38–1.56)	0.464
	<10 + ED/other	1.66 (1.06–2.60)	0.026	1.43 (0.91–2.24)	0.117
Date of pathway entry	Calendar quarter^b^	0.95 (0.92–0.98)	<0.001	0.97 (0.94–0.99)	0.008
Diagnostic time^c^	(Days)	1.00 (1.00–1.00)	0.957	−	−
TNM stage	1 (ref)	ref	ref	ref	ref
	2	3.19 (1.30–7.84)	0.011	3.12 (1.27–7.67)	0.013
	3	7.91 (3.41–18.33)	<0.001	9.14 (3.94–21.21)	<0.001
	4	60.60 (26.86–136.72)	<0.001	71.45 (31.60–161.54)	<0.001
	Incomplete	38.92 (17.00–89.20)	<0.001	26.13 (11.33–60.26)	<0.001

*CI* confidence interval, *F* female, *FIT* faecal immunochemical test, *f-Hb* faecal haemoglobin, *HR* hazard ratio, *M* male, *USC* Urgent Suspicion of Cancer.

^a^Referral to Colorectal Surgery or Gastroenterology.

^b^Calendar quarters from quarter 4 of 2018 to quarter 1 of 2023 given ascending numerical value for regression.

^c^Defined as time between date of maximum f-Hb result or referral to CRC diagnosis date recorded in cancer audit data (ICD10 codes C18, C19, C20).

Interestingly, those with f-Hb ≥10 ug/g diagnosed via ED and other specialties had the shortest diagnostic time interval (median 29 days, IQR 15–80), followed by f-Hb ≥10 ug/g and USC referral within the symptomatic pathway (median 34 days, IQR 22–70). Median times to OPD and investigation confirm that these groups are following a different diagnostic path in that those f-Hb ≥10 ug/g diagnosed outwith the symptomatic pathway had median times to cross-sectional imaging which were shorter than those to lower GI endoscopy and OPD appointment, where the USC-referred patients had an OPD and colonoscopy first approach.

Those with f-Hb ≥10 ug/g diagnosed outwith referral to colorectal surgery or gastroenterology through the symptomatic pathway had a significantly greater risk of CRC-specific death (*p* = 0.013) at univariable analysis (Fig. 2B), despite no statistically significant difference in TNM stage across the groups, and the shortest diagnostic interval. This remained the case at multivariable analysis (HR 1.47, 95% CI 1.03–2.10, *p* = 0.036) independent of TNM stage and age (Table 4).

### Sensitivity analyses

Multivariable survival analysis using those variables significantly associated with CSS in those patients with known f-Hb was then carried out using time from maximum f-Hb or referral to CRC-specific death or censor, to account for lead time bias in relation to the diagnostic interval rather than its inclusion as a variable itself (Supplementary Table 5). Again, those with f-Hb ≥10 ug/g diagnosed outwith the symptomatic pathway had poorer CSS (HR 1.48, 95% CI 1.03–2.11, *p* = 0.034) independent of TNM stage and age.

Finally, univariable CSS was compared around an f-Hb threshold of 20 ug/g (Supplementary Fig. 2B) finding no significant difference for those below compared to those above (*p* = 0.238). Given that during the study period f-Hb was used to direct referral priority using fixed values other than 20 ug/g, no analysis of pathway metrics in relation to this potential f-Hb threshold was undertaken.

## Discussion

We report a large retrospective observational study of 126,984 patients and 1453 symptomatic CRC diagnoses, over a 4 year period, within a FIT/f-Hb prioritised lower GI symptomatic pathway. As would be anticipated, there were significant differences in diagnostic time interval based on both f-Hb and referral priority. Despite this, at multivariable analysis there was no significant difference in CSS based on f-Hb alone, or between groups referred appropriately based on f-Hb results. However, referral from primary care at USC priority without an associated submitted FIT, and diagnosis outside of a f-Hb prioritised colorectal or gastroenterology pathway in those with a f-Hb ≥10 ug/g was associated with poorer colorectal cancer-specific survival adjusted for TNM stage.

Faecal haemoglobin has been reported to be associated with CRC-specific mortality in screened patients [21]. Previous reports in symptomatic patients diagnosed with CRC found a longer time to diagnosis but conflicting results in terms of CSS when those who did and did not have FIT within their diagnostic journey were compared [22, 23]. However, these studies were not performed in a specifically f-Hb-stratified pathway. Previous studies have reported that the use of f-Hb prioritised symptomatic referral pathways are associated with fewer emergency presentations [24], and a shorter time to CRC diagnosis [25]. Unlike the Tayside group, our study did find differences in TNM stage. The highest proportions of stage 3 and 4 disease were in those referred without a valid f-Hb result, and no clear linear association between stage and length of diagnostic interval in those with a valid f-Hb result.

While the proportion of patients diagnosed with CRC without a submitted FIT was 10% overall, only around 20% of these patients should have had a FIT requested according to the NHSGGC guidance of the time, with the majority having a palpable anorectal or abdominal mass, or symptoms not specific to the lower GI tract. Furthermore, there was a year-on-year reduction in the proportion referred without a f-Hb requested or a submitted but unknown/invalid result, suggesting increasing confidence in, and correct use of FIT and the pathway at all points. This group had the worst CSS and while they did not have the statistically highest rate of metastatic or locally advanced disease at diagnosis, presentation with palpable mass and atypical symptoms is more common in disease eventually treated with palliative intent [26], not captured in this study. Indeed, with the shortest diagnostic interval, this group of patients likely represents the so-called ‘sick-quick’ affected by the waiting-time paradox [27].

Given the negative implications for patients who had f-Hb ≥10 ug/g but were then diagnosed outside of the prioritised symptomatic pathway, focus must be placed on safety netting strategies at all points of the pathway from patient to primary and secondary care [28]. In relation to the former, several ongoing projects within the Cancer Research UK Test Evidence Transition (TET) Phase 2, including our own NHSGGC group, aim to optimise FIT-based diagnosis of CRC in symptomatic populations in the UK [29]. Following on from the analysis of our previous pathway described within this study, NHSGGC has recently engaged in education sessions within primary and secondary care in relation to pathway implementation changes driven by the upcoming new Scottish guidance. In addition, there has been the introduction of written safety netting communication generated by secondary care at the point of FIT resulting and delivered to patients and primary care teams to inform them of both the f-Hb and the subsequent investigation plan, the impact of which will be reported in future as part of the TET project.

Those in the most deprived SIMD quintile were over-represented in the group without a submitted FIT test both in the cohort as a whole and in those eventually diagnosed with CRC. This highlights a key concern regarding barriers to effective engagement with symptomatic pathways and stool testing in those patients from more deprived groups [30]. Similar findings have been reported across bowel cancer screening, surveillance and more recently symptomatic pathways [31, 32]. Suggested reasons include disparity in health literacy, health care system interaction, competing health priorities and economic disadvantage [33]. This is especially relevant to the NHS GGC population, half of which live in the 20% most deprived postcodes in Scotland [34], and with the lowest uptake of screening in the country [35]. Studies have also described similar findings in groups with other protected characteristics, and indeed these patients are also more likely to experience intersectional overlap with other related drivers of health inequality [36], however our cohort data did not include data on ethnicity or disability for example and so could not add to this specific area of interest.

The new Scottish Referral Guidelines For Suspected Cancer (2025) and associated Scottish national guidance document ‘Quantitative Faecal Immunochemical Testing (qFIT) 2024’ suggest the use of a f-Hb threshold of 20 ug/g to determine the need for USC referral in patients with new lower GI symptoms or asymptomatic IDA [37, 38]. The data from the present study suggest a rise in FNR from 12.6% at a threshold of 10 ug/g to 16.8% at 20 ug/g. However, the rate of CRC below both thresholds remained 0.2%, and this was in the context of a single FIT test only and without the addition of IDA status, both of which are considered within the new guidance. Univariable analysis found no significant negative impact on CSS from the higher f-Hb threshold of 20 ug/g. This result however cannot be taken as definitive as pathway groups used during the study period were in part determined by the previous f-Hb value groups which has prevented more detailed analysis in relation to referral priority and/or diagnostic delay in this setting.

The cohort described in the present study is similar to that of the Tayside and Fife groups, including all adult patients with lower GI symptoms who completed FIT or referred at USC priority through a lower GI symptomatic pathway [12, 15]. In contrast, other published data from large UK primary care symptomatic FIT cohorts from Nottingham, Oxford, Greater London, South West England and others have to a variable extent included or excluded patients based on age, NICE-defined high or low risk symptoms, or specific symptoms such as rectal bleeding [11, 39–41]. This perhaps more broadly reflects differences in NICE guidance and the Scottish Cancer Referral Guidance, with DG56 still including symptom-specific guidance based on patient age, while the current Scottish Cancer Referral Guidelines do not specify age ranges for subsets of symptoms for which FIT is indicated [8, 37, 38]. Although this does not limit the generalisability of the results of the present study, centres using pathways which strictly follow DG56 should interpret the results with caution in relation to their own population.

This study has a number of limitations due its retrospective nature. The number of CRCs in some subgroups, and subsequent events is small and some confidence intervals for CSS wide as a result. Of CRC patients alive at the time of censoring, around 11% had 2 years or less follow up from the time of diagnosis onward. However, up to 3 years was allowed between maximum f-Hb or referral and CRC diagnosis, to bring the data in line with colonoscopy-based post-test CRC literature [42], meaning that only 6% of CRC patients alive at censoring had been in the pathway for less than 2 years to censor. The sensitivity analysis, including this lead time, found similar results in terms of worse CSS in those with f-Hb ≥10ug/g diagnosed outside of colorectal surgery or gastroenterology referral. The data collection period included the COVID pandemic and recovery period during which there were even greater limits to diagnostic and therapeutic capacity. Sensitivity analysis involving specific time periods resulted in numbers of CRC deaths too small for meaningful analysis within subgroups, and as a result the date of entry into the pathway was used as a variable in survival analysis. This itself was associated with CSS, however f-Hb concentration group and referral priority remained independent.

In summary, this study provides some reassurance that FIT-based prioritisation of symptomatic referrals using guideline f-Hb threshold of 10 ug/g does not negatively impact the longer-term outcome of patients referred at lower priority appropriately. There was, however, a higher risk of CRC-specific death in those diagnosed outside the FIT prioritised symptomatic pathway. This has implications for health care providers using such pathways to maximise engagement, adherence and safety-netting strategies amongst primary care, secondary care and patients.

## Supplementary information

Supplementary Tables

Supplementary Figures

Amended STROBE checklist

Completed journal checklist

## Data Availability

Anonymised data are available on reasonable request to the authors. Access can be arranged by application to the authors and via the Glasgow SafeHaven TRE following ethical approval and the completion of mandatory information governance modules.

## Patient and public involvement

The protocol and results of this study were presented to the Glasgow Colorectal PPIE group. The authors thank the group members for their contribution to the wording and emphasis of the reporting and their ongoing help with dissemination. Dr Susanti Susanti, one of the co-authors of this paper is a PPIE representative within the CRUK TET project and the Glasgow Colorectal PPIE group with lived experience of treatment for colorectal cancer.
