# Supplementary material for: Faecal haemoglobin-based referral and investigation prioritisation is associated with colorectal cancer-specific survival in symptomatic patients: a retrospective observational study
Source: Br J Cancer. 2026 Apr 2;134(11):1580–91. doi: 10.1038/s41416-026-03378-1 (PMC13183917; doi:10.1038/s41416-026-03378-1)
Supplement: Supplementary file 1 — Supplementary Tables [file 41416_2026_3378_MOESM1_ESM.docx]

**Supplementary Tables**

Supplementary table 1: Summary of symptoms and clinical scenarios in which faecal immunochemical test (FIT) request and submission was and was not required in primary care within the NHS GGC symptomatic lower GI pathway 2018-2023

| FIT required | FIT not required |
| --- | --- |
| Rectal bleeding without obvious benign outlet cause | Non lower GI symptoms^1^ |
| New persistent change in bowel habit ≥4 weeks | Abdominal mass |
| (diarrhoea, constipation or alternating) | Rectal / anal mass or anal ulcer |
| Weight loss with lower GI symptoms | Asymptomatic iron deficiency anaemia^2^ |
| Iron deficiency anaemia^2^ with lower GI symptoms | Abnormal imaging test result |
| Persistent abdominal pain ≥4 weeks | Short lived acute and resolving symptoms (<4weeks) |
| Blood mixed through stool | Isolated thrombocytosis |
|  | Family history or genetics |
|  | Post polypectomy or post CRC surveillance |
|  | Haemorrhoids, fissure in ano, condylomata |
|  | Patient unable to complete test |
|  | No colon/rectum - ileostomy |
|  | Chronic lower GI symptoms |
|  | Rectal / pelvic organ prolapse |

CRC colorectal cancer, FIT faecal immunochemical test

1. Including dysphagia, odynophagia, vomiting, regurgitation, weight loss, fatigue without documented alteration in bowel habit or rectal bleeding
2. M: Hb <130mg/L F: Hb<120mg/L and ferritin <30ug/L

Supplementary table 2: Diagnostic performance of faecal immunochemical test at faecal haemoglobin (f-Hb) concentration thresholds of 10ug/g and 20ug/g all patients submitting faecal immunochemical test with a valid f-Hb result in primary care, or referred to lower GI secondary care services in NHS Greater and Glasgow and Clyde December 2018 to January 2023

| Total patients (n) | Total CRCs (n) | f-Hb threshold (ug/g) | Patients below threshold (n) | CRCs below threshold (n) | CRC rate below threshold  (%) | FNR below threshold  (%) | PPV above threshold (%) | NNS above threshold |
| --- | --- | --- | --- | --- | --- | --- | --- | --- |
| 115279 | 1294 | 10 | 88005 | 163 | 0.2 | 12.6 | 4.1 | 24 |
|  |  | 20 | 93101 | 218 | 0.2 | 16.8 | 4.8 | 21 |

CRC colorectal cancer, f-Hb faecal haemoglobin, FNR false negative rate, PPV positive predictive value, NNS number needed to scope

Supplementary table 3: Documented reasons for referral without submitting a faecal immunochemical test (FIT) in primary care, in patients diagnosed with colorectal cancer within 3 years of being referred to lower GI secondary care services in NHS Greater and Glasgow and Clyde December 2018 to January 2023

| Reason for non submission | n | % |
| --- | --- | --- |
| Non lower GI symptoms^1^ | 19 | 13 |
| Abdominal mass | 8 | 6 |
| Rectal / anal mass or anal ulcer | 29 | 20 |
| Asymptomatic iron deficiency anaemia^2^ | 28 | 20 |
| Abnormal imaging test result | 7 | 5 |
| Recent positive bowel screening FIT | 9 | 6 |
| Requested but not returned by patient | 11 | 8 |
| Not requested despite appropriate symptoms | 29 | 21 |
| Other | 1 | 1 |

FIT faecal immunochemical test

1. Including dysphagia, odynophagia, vomiting, regurgitation, weight loss, fatigue without documented alteration in bowel habit or rectal bleeding
2. M: Hb <130mg/L F: Hb<120mg/L and ferritin <30ug/L

Supplementary table 4: Patient characteristics, pathway process measurement, stage and survival in patients diagnosed with colorectal cancer within 3 years of submitting faecal immunochemical test in primary care, or being referred to lower GI secondary care services in NHS Greater and Glasgow and Clyde December 2018 to January 2023

| f-Hb (ug/g) |  | ≥10 | | | <10 | | | p | p adj |
| --- | --- | --- | --- | --- | --- | --- | --- | --- | --- |
| Referral^1^ priority |  | **USC** | **Urgent/Routine** | **ED / other speciality** | **USC** | **Urgent/Routine** | **ED / other specialty** |  |  |
| Patients | n(%) | 857 (66) | 186 (14) | 88 (7) | 59 (5) | 36 (3) | 68 (5) | - |  |
|  |  |  |  |  |  |  |  |  |  |
| Patient characteristics | (% within rows) |  |  |  |  |  |  |  |  |
| Age (yrs) | Median (IQR) | 71 (61-80) | 67 (57-78) | 71 (65-81) | 67 (61-80) | 69 (62-67) | 72 (62-80) | 0.050 | 0.056 |
| Sex n(%) | M | 517 (70) | 100 (13) | 45 (6) | 30 (4) | 14 (2) | 36 (5) | 0.035 | 0.044 |
|  | F | 340 (62) | 86 (16) | 43 (8) | 29 (5) | 22 (3) | 32 (6) |  |  |
| SIMD quintile n(%) | 1 (most deprived) | 262 (65) | 66 (16) | 19 (5) | 20 (5) | 16 (4) | 20 (5) | 0.012 | 0.017 |
|  | 2 | 161 (65) | 27 (11) | 17 (7) | 19 (8) | 5 (2) | 19 (8) |  |  |
|  | 3 | 105 (67) | 29 (18) | 10 (6) | 5 (3) | 1 (1) | 8 (5) |  |  |
|  | 4 | 119 (63) | 34 (18) | 16 (9) | 4 (2) | 8 (4) | 7 (4) |  |  |
|  | 5 (least deprived) | 209 (70) | 30 (10) | 26 (9) | 11 (4) | 6 (2) | 14 (5) |  |  |
| Year of entry to pathway n(%) | 2019 | 113 (57) | 52 (26) | 9 (4) | 7 (4) | 10 (5) | 7 (4) | <0.001 | <0.001 |
|  | 2020 | 178 (62) | 43 (15) | 25 (9) | 15 (5) | 7 (3) | 18 (6) |  |  |
|  | 2021 | 263 (68) | 48 (12) | 21 (5) | 21 (5) | 14 (4) | 22 (6) |  |  |
|  | 2022 | 303 (72) | 43 (10) | 33 (8) | 16 (4) | 5 (1) | 21 (5) |  |  |
| Anaemia n(%)^1^ | No | 441 (56) | 110 (64) | 29 (38) | 35 (60) | 17 (54) | 32 (51) | 0.051 | 0.055 |
|  | Yes – not IDA^2^ | 137 (17) | 23 (14) | 21 (27) | 9 (16) | 7 (23) | 14 (22) |  |  |
|  | Yes – IDA^2^ | 218 (27) | 38 (22) | 27 (35) | 14 (24) | 7 (23) | 17 (27) |  |  |
|  |  |  |  |  |  |  |  |  |  |
| Pathway characteristics |  |  |  |  |  |  |  |  |  |
| Time to OPD (days)^3^ | Median (IQR) | 27 (11-57) | 39 (15-70) | 33 (13-57) | 28 (13-48) | 47 (12-114) | 127 (41-372) | <0.001 | <0.001 |
| Time to scope (days)^3^ | Median (IQR) | 31 (22-59) | 46 (27-100) | 32 (21-82) | 185 (57-466) | 168 (47-333) | 513 (175-744) | <0.001 | <0.001 |
| Time to CT scan^4^ (days)^3^ | Median (IQR) | 48 (33-83) | 65 (42-98) | 31 (9-85) | 79 (43-223) | 108 (36-267) | 161 (33-603) | <0.001 | <0.001 |
| Total diagnostic interval (days)^5^ | Median (IQR) | 34 (22-70) | 46 (28-100) | 29 (15-80) | 121 (46-324) | 128 (56-571) | 280 (84-657) | <0.001 | <0.001 |
|  |  |  |  |  |  |  |  |  |  |
| Tumour characteristics | (% within columns) |  |  |  |  |  |  |  |  |
| TNM stage n(%) | 1 | 151 (18) | 45 (24) | 13 (15) | 13 (22) | 11 (31) | 10 (15) | 0.226 | 0.226 |
|  | 2 | 210 (24) | 40 (22) | 17 (19) | 9 (15) | 3 (8) | 18 (26) |  |  |
|  | 3 | 219 (26) | 42 (23) | 20 (23) | 14 (24) | 9 (25) | 12 (18) |  |  |
|  | 4 | 176 (20) | 38 (20) | 22 (25) | 17 (29) | 9 (25) | 20 (29) |  |  |
|  | Incomplete | 101 (12) | 21 (11) | 16 (18) | 6 (10) | 4 (11) | 8 (12) |  |  |
| Tumour location n(%) | Right colon | 283 (32) | 66 (36) | 49 (55) | 29 (49) | 17 (47) | 40 (59) | <0.001 | <0.001 |
|  | Left colon | 260 (31) | 63 (33) | 22 (27) | 15 (25) | 8 (22) | 19 (28) |  |  |
|  | Rectum | 312 (36) | 57 (31) | 16 (18) | 15 (25) | 11 (31) | 8 (12) |  |  |
|  | Unspecified | 2 (1) | 0 (0) | 1 (1) | 0 (0) | 0 (0) | 1 (1) |  |  |

CRC colorectal cancer, CSS cancer specific survival, CT computed tomography scan, ED Emergency Department, F female, f-Hb faecal haemoglobin, FIT faecal immunochemical test, IDA iron deficiency anaemia, IQR interquartile range, M male, OPD outpatient department, SE standard error, SIMD Scottish Index of Multiple Deprivation, USC Urgent Suspicion of Cancer

1. M: Hb <130mg/L F: Hb<120mg/L
2. Anaemia and ferritin <30ug/L
3. Defined as time in days between date of maximum f-Hb result or referral and date of OPD or investigation appointment
4. CT pneumocolon, CT thorax and abdomen and pelvis, or CT abdomen and pelvis
5. Defined as time between date of maximum f-Hb result or referral to CRC diagnosis date recorded in cancer audit data (ICD10 codes C18, C19, C20)

Supplementary table 5: Multivariable Cox regression of Cancer specific survival^1^ - in patients diagnosed with colorectal cancer within 3 years of submitting faecal immunochemical test in primary care, or being referred to lower GI secondary care services in NHS Greater and Glasgow and Clyde December 2018 to January 2023

|  |  | Multivariable HR (95% CI) | p |
| --- | --- | --- | --- |
| Age | (Years) | 1.03 (1.02-1.04) | <0.001 |
| Max f-Hb (ug/g) and | ≥10 + USC | ref | ref |
| Referral^2^ priority | ≥10 + Urgent/Routine | 1.21 (0.90-1.64) | 0.201 |
|  | ≥10 + ED/other | 1.48 (1.03-2.11) | 0.034 |
|  | <10 + USC | 0.90 (0.54-1.49) | 0.670 |
|  | <10 + Urgent/Routine | 0.72 (0.35-1.46) | 0.363 |
|  | <10 + ED/other | 1.11 (0.71-1.74 ) | 0.646 |
| Date of pathway entry | Calendar quarter^3^ | 0.97 (0.94-0.99) | 0.013 |
| TNM stage | 1 (ref) | ref | ref |
|  | 2 | 3.15 (1.28-7.73) | 0.012 |
|  | 3 | 9.16 (3.95-21.25) | <0.001 |
|  | 4 | 68.29 (30.20-154.39) | <0.001 |
|  | Incomplete | 25.00 (10.84-57.65) | <0.001 |

CI confidence interval, f-Hb faecal haemoglobin, HR hazard ratio, USC Urgent Suspicion of Cancer

1. Defined as time between date of maximum f-Hb result or referral to CRC death recorded in cancer audit data (ICD10 codes C18, C19, C20) or censor
2. Referral to Colorectal Surgery or Gastroenterology
3. Calendar quarters from quarter 4 of 2018 to quarter 1 of 2023 given ascending numerical value for regression
