# Supplementary material for: Faecal haemoglobin-based referral and investigation prioritisation is associated with colorectal cancer-specific survival in symptomatic patients: a retrospective observational study
Source: Br J Cancer. 2026 Apr 2;134(11):1580–91. doi: 10.1038/s41416-026-03378-1 (PMC13183917; doi:10.1038/s41416-026-03378-1)
Supplement: Supplementary file 2 — Supplementary Figures [file 41416_2026_3378_MOESM2_ESM.docx]

**
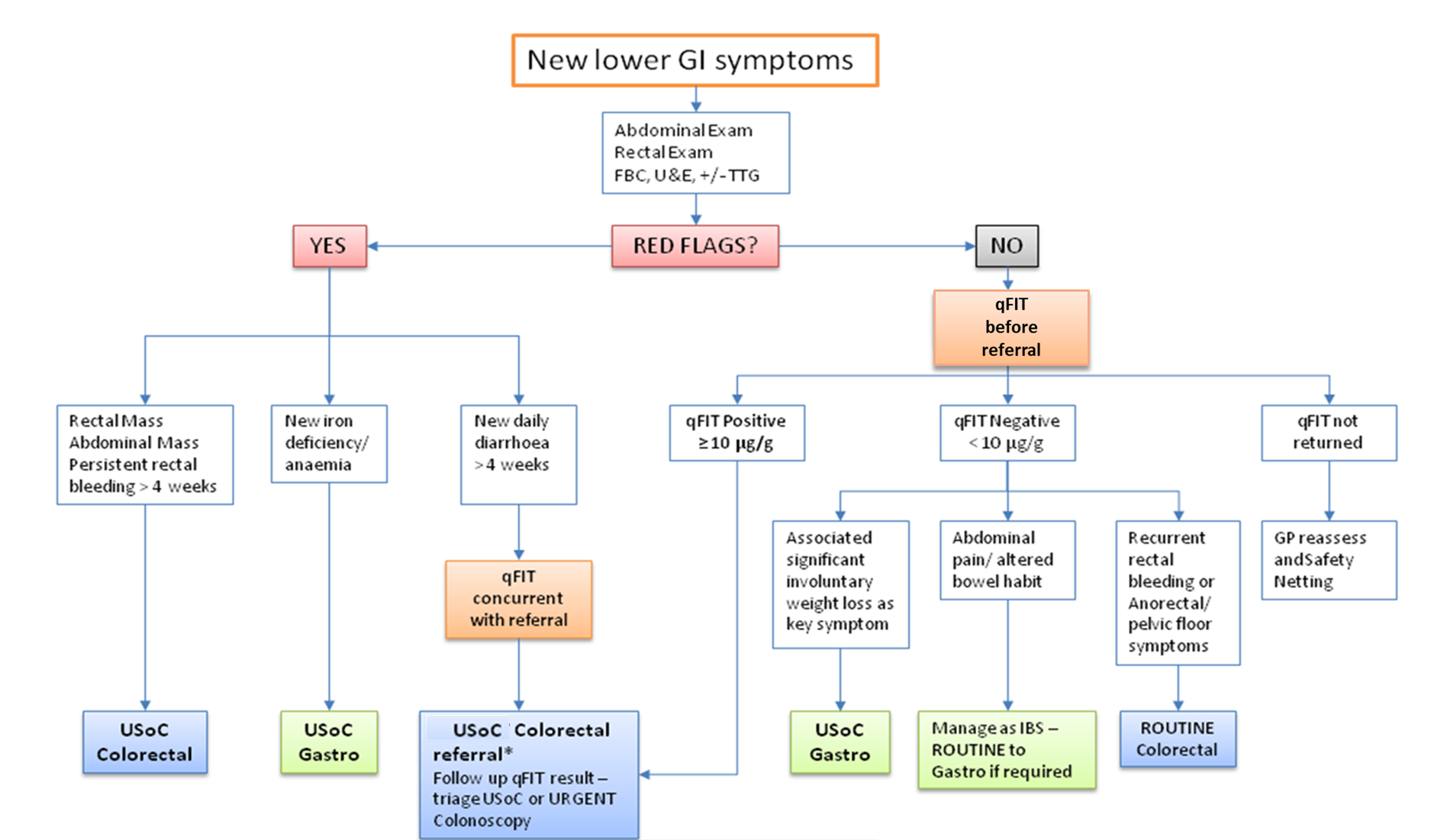
Supplementary Figures**

Supplementary Figure 1: Schematic of lower GI symptomatic pathway NHS GGC 2018 -2023. FBC full blood count, qFIT quantitative faecal immunochemical test, TTG tissue transglutaminase, U&E Urea and electrolytes, USoC Urgent suspicion of cancer


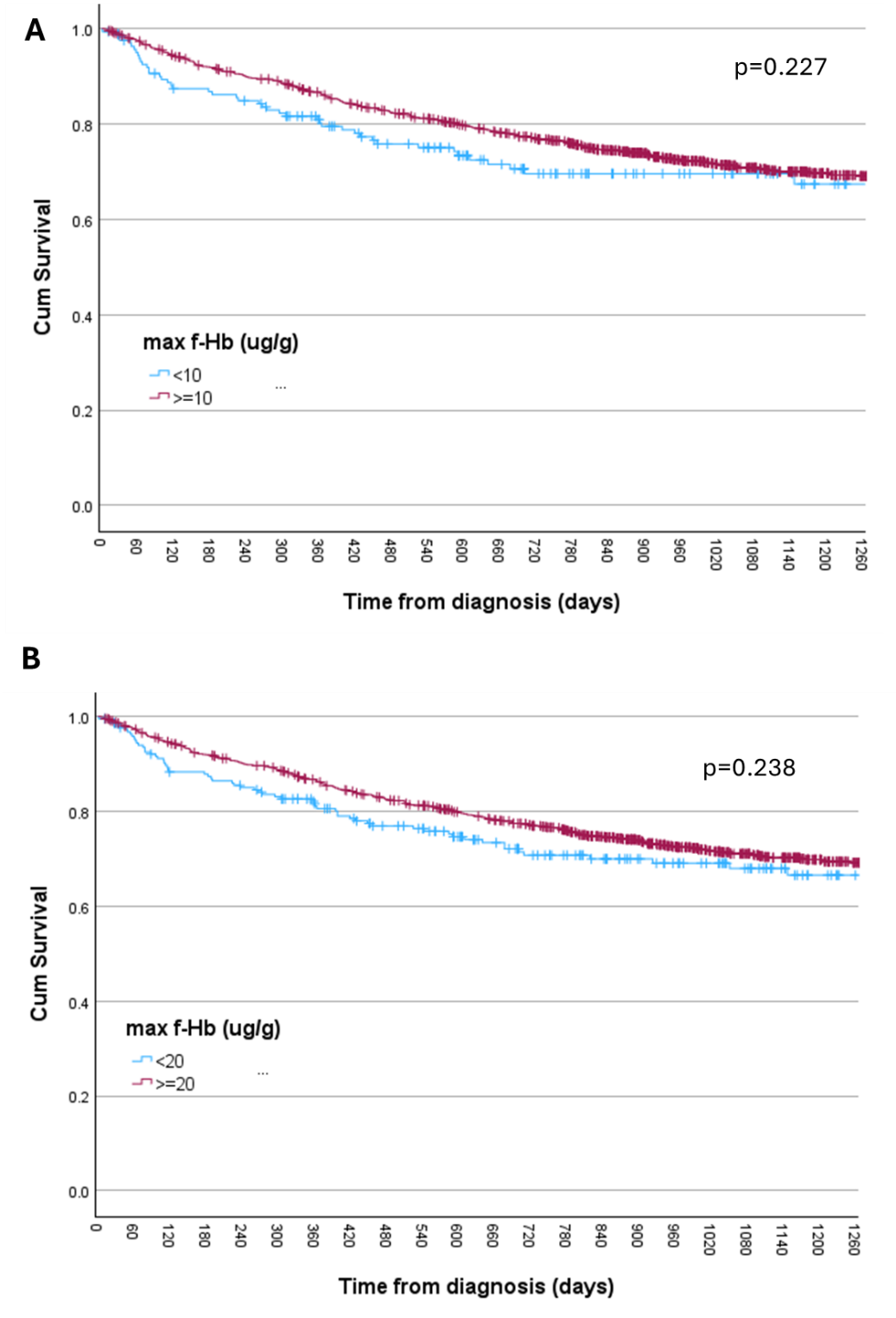


Supplementary Figure 2: Kaplan Meier curve of colorectal cancer (CRC) specific survival (CSS) defined by ICD10 codes 18, 19 and 20, in patients with a valid faecal haemoglobin (f-Hb) result (n=1294), grouped by f-Hb concentration threshold of **A)** 10ug/g (p=0.227) and **B)** 20ug/g (p=0.238
